# Hemorrhagic Malarial Fever, and Our Journal

**Published:** 1877-05-20

**Authors:** 


### HEMORRHAGIC MALARIAL FEVER, AND OUR JOURNAL.

Dr. L. A. Guild, of Georgia, has promised to send us a new and successful treatment for that formidable malady known as *Hemorrhagic Malarial Fever*. We hope to have it in time for our next issue.

He says of our journal: "I have been a constant reader of your journal *with four other medical periodicals*, and regard the SOUTHERN MEDICAL RECORD as the best for the general practitioner. While I find much more display and extravagance of get up in the northern journals, and a great deal of *reading* matter pertaining to hospitals, syphilitic affections, aneurisms, elaborate theories and prosy statistical reports, and a vast deal about diseases which the village and country doctor never sees, I find in your journal pithy and pointed communications about diseases which are incident to my own section, and which I almost daily encounter; and in connection therewith I find practical suggestions, and new and valuable formulae, which I can make, and have made available in my practice. I can point out in each issue of your journal receipes which I would not be deprived of for the price of subscription. This much I have said in no spirit of flattery, but because it is the truth, and because I feel that you ought to be encouraged and sustained by the profession. I hope soon to send you a subscriber. * * *

[We thank our friend for the high compliment he has thought fit to give us. He is not alone in his high estimation of the RECORD; we have a large number of testimonials equally complimentary, to publish which would be agreeable to our vanity, but would encroach too much upon our space.]

